# Added value of whole‐exome and RNA sequencing in advanced and refractory cancer patients with no molecular‐based treatment recommendation based on a 90‐gene panel

**DOI:** 10.1002/cam4.7115

**Published:** 2024-03-30

**Authors:** Armelle Dufresne, Valéry Attignon, Anthony Ferrari, Laurie Tonon, Sandrine Boyault, Séverine Tabone‐Eglinger, Philippe Cassier, Olivier Trédan, Nadège Corradini, Armelle Vinceneux, Aurélie Swalduz, Alain Viari, Sylvie Chabaud, David Pérol, Jean Yves Blay, Pierre Saintigny

**Affiliations:** ^1^ Department of Medical Oncology Centre Léon Bérard Lyon France; ^2^ Platform of Cancer Genomics Centre Léon Bérard Lyon France; ^3^ Platform of Bioinformatics Gilles‐Thomas Centre Léon Bérard Lyon France; ^4^ Biobank Centre Léon Bérard Lyon France; ^5^ Department of Pediatric Oncology, Institute of Pediatric Hematology and Oncology Centre Leon Bérard Lyon France; ^6^ Department of Clinical Research Centre Léon Bérard Lyon France; ^7^ Univ Lyon, Claude Bernard Lyon 1 University, INSERM 1052, CNRS 5286, Centre Léon Bérard Cancer Research Center of Lyon Lyon France

**Keywords:** cancer biology, cancer management, gene panel, molecular tumor board, precision oncology, RNA‐sequencing, targeted therapy, whole exome sequencing

## Abstract

**Introduction:**

The objective was to determine the added value of comprehensive molecular profile by whole‐exome and RNA sequencing (WES/RNA‐Seq) in advanced and refractory cancer patients who had no molecular‐based treatment recommendation (MBTR) based on a more limited targeted gene panel (TGP) plus array‐based comparative genomic hybridization (aCGH).

**Materials and Methods:**

In this retrospective analysis, we selected 50 patients previously included in the PROFILER trial (NCT01774409) for which no MBT could be recommended based on a targeted 90‐gene panel and aCGH. For each patient, the frozen tumor sample mirroring the FFPE sample used for TGP/aCGH analysis were processed for WES and RNA‐Seq. Data from TGP/aCGH were reanalyzed, and together with WES/RNA‐Seq, findings were simultaneously discussed at a new molecular tumor board (MTB).

**Results:**

After exclusion of variants of unknown significance, a total of 167 somatic molecular alterations were identified in 50 patients (median: 3 [1–10]). Out of these 167 relevant molecular alterations, 51 (31%) were common to both TGP/aCGH and WES/RNA‐Seq, 19 (11%) were identified by the TGP/aCGH only and 97 (58%) were identified by WES/RNA‐Seq only, including two fusion transcripts in two patients. A MBTR was provided in 4/50 (8%) patients using the information from TGP/aCGH versus 9/50 (18%) patients using WES/RNA‐Seq findings. Three patients had similar recommendations based on TGP/aCGH and WES/RNA‐Seq.

**Conclusions:**

In advanced and refractory cancer patients in whom no MBTR was recommended from TGP/aCGH, WES/RNA‐Seq allowed to identify more alterations which may in turn, in a limited fraction of patients, lead to new MBTR.

## 1 INTRODUCTION

The concept of tumor‐agnostic precision oncology is now integrated in routine for a limited set of somatic molecular alterations.^1^, ^2^, ^3^ In studies assessing the throughput of tumor molecular analysis for patients with advanced solid tumor, the proportion of patients treated with molecular‐based therapy ranged from 6% to 26%.^4^, ^5^, ^6^, ^7^ This low proportion may have prevented these trials to conclude on the benefit of agnostic precision oncology.^8^, ^9^, ^10^ In contrast, several meta‐analysis studies reported a significant benefit of a genomic‐driven personalized approach to drive patients in Phase 1 and 2 trials. Extending the molecular analysis to the entire exome may increase the proportion of actionable molecular alterations, of molecular‐based treatment recommendations (MBTR) and eventually, of treated patients.

To determine to which extent a whole‐exome and RNA sequencing (WES/RNA‐Seq) analysis increases the proportion of patients with MBTR, a retrospective analysis was conducted in a subset of 50 patients included in PROFILER study (molecular screening by TGP/aCGH to select molecular‐based recommended therapies for metastatic cancer patients). For each cases, there were available germ line DNA and fresh frozen tumor mirroring the FFPE sample used for Tumor Gene Panel and array‐based Comparative Genomic Hybridization (TGP/aCGH) analysis and had no MBTR based on the TGP/aCGH.^7^

## 2 MATERIALS AND METHODS

### 2.1 Patients, sample qualification, and molecular analysis

The study was conducted at Centre Léon Bérard was approved on 2/2/2018 by the institutional review board (Ethics Committee of Lyon Sud‐Est IV) and was conducted in compliance with the Declaration of Helsinki and Good Clinical Practice guidelines.

**FIGURE 1:**
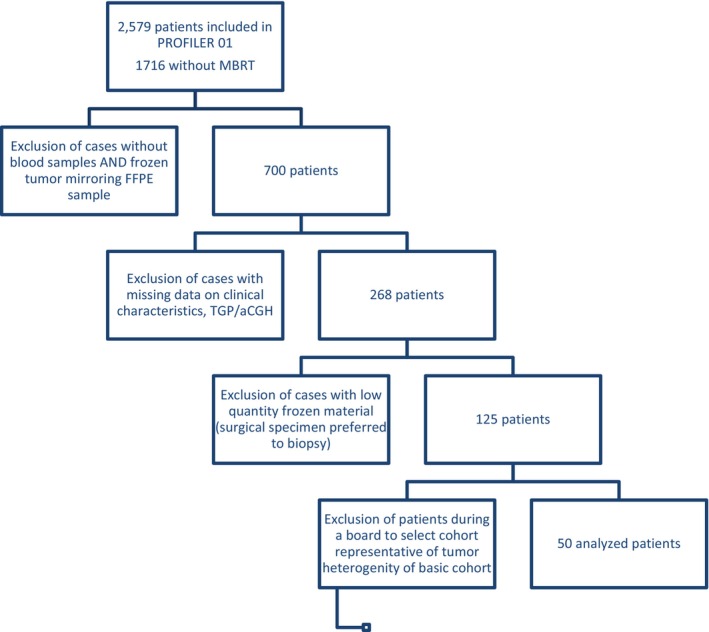
Consort diagram of the selection of studied population from the PROFILER01 clinical trial.

We retrospectively selected 50 patients among the 2,579 patients included in the previously reported PROFILER molecular screening program (NCT01774409) who had no MBTR based on the TGP/aCGH during the course of the trial and for whom a fresh frozen tumor mirroring the FFPE sample together with germ line DNA was available in Centre Léon Bérard certified Biobank (BB‐0033‐00050).^7^ Gene list of the TGP is provided in Table S1. Fresh frozen surgically resected tumor specimens mirroring the FFPE sample were evaluated by an experienced pathologist for tumor cell content ≥30% was required. The first 50 cases achieving those criteria were included in the study. Each patient included in PROFILER provided written informed consent to participate in the study and use of his or her tumor sample. Figure 1 presents the consort diagram of the selection of studied population from the whole cohort of the PROFILER01 clinical trial.

The molecular analysis conducted in PROFILER trial was reported elsewhere.^7^ Details on WES/RNA‐Seq and bioinformatics analysis are provided in Methods S1.

### 2.2 Variants interpretation and treatment recommendation

Analysis pipelines are regularly updated overtime, TGP raw data for the 50 selected patients were thus reanalyzed and a new report issued. Both the TGP and WES/RNA‐Seq reports were presented at the Molecular Tumor Board (MTB). The interpretation of somatic single nucleotide variants (SNV) was focused on their clinical impacts and categorized into five TIERs according to the ESMO Scale for Clinical Actionability of molecular Targets (ESCAT) classification^11^ (Figure S1). MTB presentation was done at the same time to ensure similar treatment options for both tests.

### 2.3 Statistical analysis

Statistical analyses were conducted with SPSS 23.0 package (IBM, Paris, France). The proportion of variants in each Tier of the ESCAT classification identified with TGP/aCGH versus WES/RNA‐Seq was compared using a Fisher's exact test. A *p* value of 0.05 was considered significant.

## 3 RESULTS

The cohort of 50 patients included 14 different histological subtypes of cancer (Table 1). They were comparable to the overall population of the PROFILER study.

**TABLE 1:** Clinical and pathological characteristics of the patients included in the study.

	Subset of patients included in the study (*N* = 50)
Age at diagnosis	
Median (range)	54.0 (21.0–81.0)
Gender	
Male	22 (44%)
Female	28 (66%)
ECOG‐PS	
Missing data	5 (10%)
0	12 (24%)
1	31 (%)
≥2	2 (4%)
Delay from the date of diagnosis of noncurable disease to inclusion (years)	
Median (range)	1.0 (0–11.4)
Primary tumor site	
Breast	9 (18%)
Ovary	8 (16%)
Colorectal	6 (12%)
Sarcoma	6 (12%)
Head and neck	5 (10%)
Other	16 (32%)

Abbreviation: MBRT, molecular‐based recommended therapies.

After exclusion of 4 (TGP) and 9619 (WES) variants of unknown significance, TGP and WES identified 52 SNVs and 121 indels. Respectively 70 and 148 molecular alterations including SNVs (*n* = 135, 80%), CNVs (*n* = 29, 17%), one indel (*n* = 1, <1%), one tumor mutational burden (TMB) >10 mutations per megabase (median TMB: 1, range: 0–24.5), and fusion transcripts (*n* = 2, 1%–2%) were reported by the biologist with TGP/aCGH (median per patient 1, range 0–6) and WES/RNA‐Seq (median per patient 2, range 0–8). Out of 167 molecular alterations (Table S2), 51 (30%) were common to both TGP/aCGH and WES/RNA‐Seq, 19 (11%) were identified by the TGP/aCGH only, and 97 (58%) were identified by WES/RNA‐Seq only. Among the latest, two patients were found with a fusion gene by RNAseq (COL1A1::PDGFB or PAX5::FOXP1) that were already known from the initial diagnostic workup. More ESCAT TIER IV and X molecular alterations were identified by WES/RNA‐Seq (Table 2).

**TABLE 2:** Frequency of molecular alterations identified with the 90‐gene TGP/aCGH or WES/RNA‐Seq the classified according to ESCAT.^11^

	90‐gene TGP/aCGH	WES/RNA‐Seq
TIER I	3 (4%)	6 (4%)
TIER II	13 (19%)	17 (12%)
TIER III	33 (47%)	44 (30%)
TIER IV	10 (14%)	33 (22%)
TIER X	11 (16%)	48 (32%)
Total	70 (100%)	148 (100%)

*Note*: Proportion of variants in each Tier of the ESCAT classification identified with TGP/aCGH versus WES/RNA‐Seq was compared using a Fisher's exact test (*p* = 0.0154).

**FIGURE 2:**
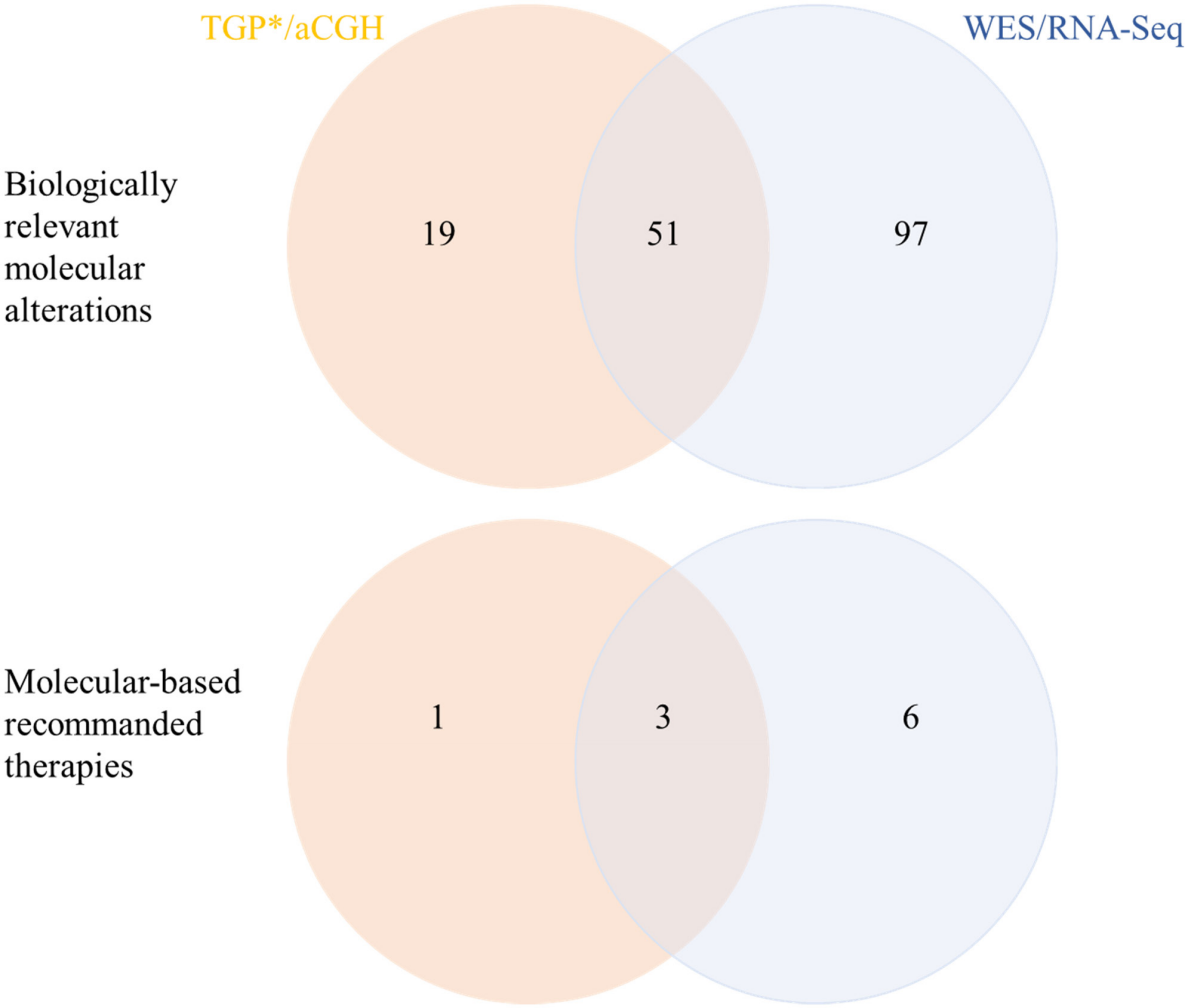
Venn diagram of biologically relevant molecular alterations identified with TGP/aCGH versus WES/RNA‐Seq in advanced and refractory patients with no molecular‐based recommended therapy. *TGP raw data for the 50 selected patients were reanalyzed, and a new report was issued. Both the TGP/aCGH and WES/RNA‐Seq reports were presented at the MTB.

Whether MBTR differed when they were based on TGP/aCGH versus WES/RNA‐Seq was discussed at the MTB (Figure 2). A MBTR was recommended in 4/50 (8%) patients using the information from TGP/aCGH versus 9/50 (18%) patients using WES/RNA‐Seq findings. Three patients had similar recommendations (PI3K/Akt/mTOR inhibitor and KRAS G12C inhibitor in two and one cases, respectively) based on either TGP/aCGH or WES/RNA‐Seq (Figure 2). The presence of these four cases can be explained by the fact that all cases were discussed in molecular tumor board to compare both panel at the same time (second discussion for old cases). The six MBTR exclusively provided by WES/RNA‐Seq were (1) a PKC inhibitor for a choroidal melanoma with a GNAQ SNV (not included in the TGP panel), (2) a KIT inhibitor for a gastrointestinal stromal tumor with a KIT D820E mutation (region not covered by TGP), (3) an immune therapy based on a high TMB on WES (not available on TGP) for a malignancy of unknown origin, (4) a PARP inhibitor based on a BRCA loss not identified by TGP for a serous ovarian cancer and (5) and (6) were recommended a PI3K/Akt/mTOR inhibitor based on a PI3K p.N345K mutation not identified by TGP for an invasive ductal carcinoma and a PTEN p.M1L for a pyloric *adenocarcinoma*.

## 4 DISCUSSION

Molecular analysis by WES/RNA‐Seq is now available in routine for diagnosis and theranostic purposes to increase the rate of MBTR for patients with advanced cancer. To our knowledge, this is the first report comparing the percentage of candidate patients for a MBTR using both TGP/aCGH and WES/RNA‐Seq available in all patients. As expected, WES/RNA‐Seq led to the identification of more molecular alterations but most were not used for MBTR in the absence of documented clinical significance. However, a numerically higher rate of MBTR was recommended compared to TGP/aCGH. The translation of these recommendations into clinical benefit for the patients remains to be determined. Only 51 (30%) molecular alterations were common to both TGP/aCGH and WES/RNA‐Seq. These discrepancies may be explained by tumor heterogeneity: Although the same tumor was analyzed, nucleic acids were extracted from a FFPE sample (TGP/aCGH) and from a frozen sample (WES/RNA‐Seq). As expected, some molecular alterations were missed by TGP because genes were not included in the panel, or because no fusion can be studied with TGP. Also, gene expression data and expression profile were not included in discussion for MBTR recommendation but represent promising biomarkers. Discrepancies between TGP and WES/RNAseq may also be related to lower sequencing depth (false negatives), and to the subtraction of constitutional variants (true negatives).

Results of the prospective randomized PROFILER02 trial were presented in ASCO 2022: Compared to TGP/aCGH panel, larger NGS panel led to increase MBTR from 5% to 19.8%, very similar to our results from 8% to 18%.^12^

Other groups reported impressive rate of 31.8% and 46% of patients treated with molecular‐based therapy after extensive genomic analysis.^13^, ^14^ However, the definition of “actionability” of a given molecular alteration remains unclear.^15^ In the study, we selected patients who were given no recommendation in the course of the trial based on TGP/aCGH, possibly explaining the low rate of patients with MBTR based on WES/RNA‐Seq [9/50 (18%)].

## 5 CONCLUSION

In this work, WES/RNA‐Seq analysis resulted in a significantly superior but modest improvement of the number of MBTR compared to TGP/aCGH. Discrepancies were observed between the two tests, owing possibly to sample quality bias, and subclonal analysis. As more knowledge is gained on the significance of individual and combined mutations based on WES/RNA‐Seq, a careful clinical evaluation of the utility of WES/RNA‐Seq for the management of cancer patients with advanced and refractory disease must be undertaken to further compare the utility of narrow panels versus broader but more expensive approaches.

## AUTHOR CONTRIBUTIONS

**Armelle Dufresne:** Conceptualization (equal); data curation (equal); formal analysis (equal); investigation (equal); methodology (equal); supervision (equal); validation (equal); writing – original draft (equal); writing – review and editing (equal). **Valéry Attignon:** Conceptualization (equal); data curation (equal); formal analysis (equal); funding acquisition (equal); methodology (equal); software (equal); visualization (equal); writing – original draft (equal); writing – review and editing (equal). **anthony ferrari:** Conceptualization (equal); data curation (equal); formal analysis (equal); funding acquisition (equal); methodology (equal); software (equal); visualization (equal); writing – original draft (equal); writing – review and editing (equal). **laurie tonon:** Conceptualization (equal); data curation (equal); formal analysis (equal); funding acquisition (equal); methodology (equal); software (equal); visualization (equal); writing – original draft (equal); writing – review and editing (equal). **sandrine boyault:** Conceptualization (equal); data curation (equal); formal analysis (equal); funding acquisition (equal); methodology (equal); software (equal); visualization (equal); writing – original draft (equal); writing – review and editing (equal). **Séverine Tabone‐Eglinger:** Resources (equal); writing – review and editing (equal). **philippe cassier:** Investigation (equal); validation (equal); writing – review and editing (equal). **Olivier Trédan:** Investigation (equal); validation (equal); writing – review and editing (equal). **Nadège Corradini:** Investigation (equal); validation (equal); writing – review and editing (equal). **armelle vinceneux:** Investigation (equal); validation (equal); writing – review and editing (equal). **Aurélie Swalduz:** Investigation (equal); validation (equal); writing – review and editing (equal). **alain viari:** Investigation (equal); methodology (equal); supervision (equal); validation (equal); writing – review and editing (equal). **SYLVIE CHABAUD:** Formal analysis (equal); methodology (equal); writing – review and editing (equal). **David Pérol:** Methodology (equal); supervision (equal); validation (equal); writing – review and editing (equal). **Jean Yves Blay:** Conceptualization (equal); methodology (equal); supervision (equal); validation (equal); writing – original draft (equal); writing – review and editing (equal). **Pierre Saintigny:** Conceptualization (equal); formal analysis (equal); funding acquisition (equal); investigation (equal); methodology (equal); project administration (equal); supervision (equal); validation (equal); writing – original draft (equal); writing – review and editing (equal).

## FUNDING INFORMATION

This work was supported by funding from the Integrated Cancer Research Site LYriCAN (INCa‐DGOS‐Inserm_12563), NetSARC (INCA & DGOS), InterSARC (INCA), LabEx DEvweCAN (ANR‐10‐LABX 0061), PIA Institut Convergence Francois Rabelais PLAsCAN (PLASCAN, 17‐CONV‐0002), Fondation ARC contre le Cancer, La Ligue contre le Cancer (Canopée), and EURACAN (EC 739521).

## IMPACT STATEMENT

Comprehensive tumor molecular profile by WES/RNA‐Seq increased the number of MBTR as compared to a TGP/aCGH screening to only a minority of patients. As more knowledge is gained on the significance of mutations based on WES/RNA‐Seq, a careful clinical evaluation of the utility of WES/RNA‐Seq for the management of cancer patients with advanced and refractory disease must be undertaken to further compare the utility of narrow panels versus broader but more expensive approaches.

## Supporting information


Figure S1:

Data S1.

Table S1:

Table S2:

## Data Availability

The data that support the findings of this study are available on request from the corresponding author, Dr Pierre Saintigny.
