# Supplementary material for: Added value of whole‐exome and RNA sequencing in advanced and refractory cancer patients with no molecular‐based treatment recommendation based on a 90‐gene panel
Source: Cancer Med. 2024 Mar 30;13(7):e7115. doi: 10.1002/cam4.7115 (PMC10980928; doi:10.1002/cam4.7115)
Supplement: Supplementary file 1 — Figure S1: [file CAM4-13-e7115-s003.docx]

**Supplementary Figure 1: Bioinformatic workflow to classify molecular alterations according to ESCAT [1]**

**
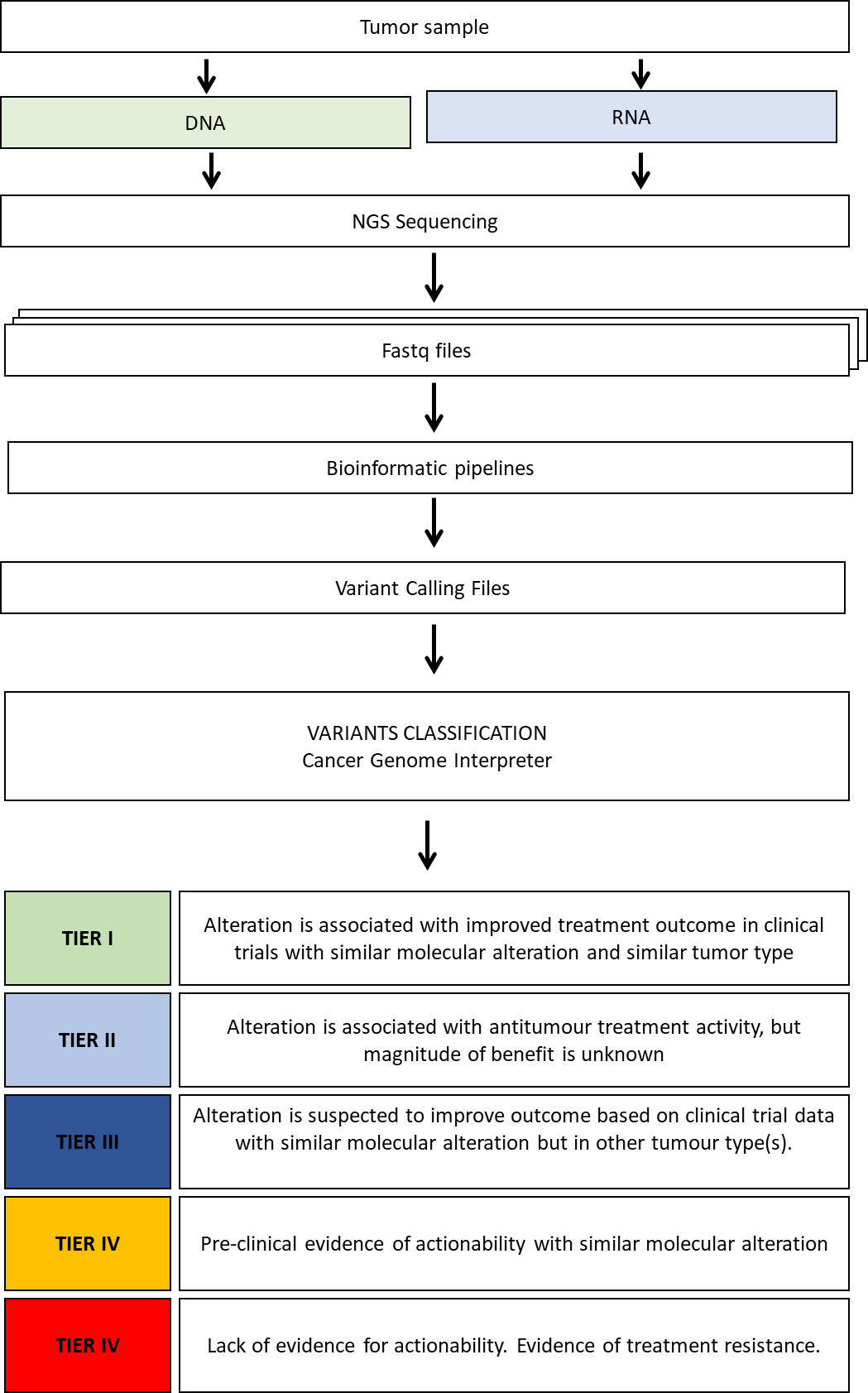
**

1. Mateo, J., et al., *A framework to rank genomic alterations as targets for cancer precision medicine: the ESMO Scale for Clinical Actionability of molecular Targets (ESCAT).* Ann Oncol, 2018. **29**(9): p. 1895-1902.
