# Supplementary material for: Added value of whole‐exome and RNA sequencing in advanced and refractory cancer patients with no molecular‐based treatment recommendation based on a 90‐gene panel
Source: Cancer Med. 2024 Mar 30;13(7):e7115. doi: 10.1002/cam4.7115 (PMC10980928; doi:10.1002/cam4.7115)
Supplement: Supplementary file 3 — Table S1: [file CAM4-13-e7115-s002.docx]

**Supplementary Table 1: Gene list of the targeted 90-gene panel used in the PROFILER study [1]**

| **Gene** | **Transcript** | **Gene** | **Transcript** | **Gene** | **Transcript** |
| --- | --- | --- | --- | --- | --- |
| **AKT1** | NM_005163 | **DDR1** | NM_001202523 | **IDH2** | NM_002168 |
| **AKT2** | NM_001626 | **DDR2** | NM_006182 | **IGF1R** | NM_000875 |
| **ALK** | NM_004304 | **EGFR** | NM_005228 | **JAK3** | NM_000215 |
| **APC** | NM_001127511 | **ERBB2** | NM_004448 | **KDR** | NM_002253 |
| **ARID1A** | NM_006015 | **ERBB4** | NM_005235 | **KIT** | NM_000222 |
| **ARID1B** | NM_020732 | **EZH2** | NM_001203247 | **KRAS** | NM_004985 |
| **ATM** | NM_000051 | **FGFR1** | NM_023106 | **MAP2K1** | NM_002755 |
| **ATR** | NM_001184 | **FGFR2** | NM_000141 | **MDM2** | NM_002392 |
| **AXL** | NM_021913 | **FGFR3** | NM_000142 | **MERTK** | NM_006343 |
| **BRAF** | NM_004333 | **FGFR4** | NM_002011 | **MET** | NM_001127500 |
| **BRCA1** | NM_007294 | **FLT1** | NM_002019 | **MLH1** | NM_000249 |
| **BRCA2** | NM_000059 | **FLT3** | NM_004119 | **MSH2** | NM_000251 |
| **CDKN2A** | NM_000077 | **FLT4** | NM_182925 | **MSH6** | NM_000179 |
| **CSF1R** | NM_005211 | **HRAS** | NM_005343 | **MST1R** | NM_001244937 |
| **DDB2** | NM_000107 | **IDH1** | NM_005896 | **MTOR** | NM_004958 |
|  |  |  |  |  |  |
| **Gene** | **Transcript** | **Gene** | **Transcript** | **Gene** | **Transcript** |
| **MUSK** | NM_001166280 | **POLE** | NM_006231 | **SDHC** | NM_001035511 |
| **NRAS** | NM_002524 | **PTCH1** | NM_001083603 | **SDHD** | NM_003002 |
| **NTRK1** | NM_002529 | **PTEN** | NM_000314 | **SMARCA2** | NM_003070 |
| **NTRK2** | NM_006180 | **RAD51C** | NM_058216 | **SMARCA4** | NM_001128849 |
| **NTRK3** | NM_001012338 | **RAD51D** | NM_001142571 | **SMARCB1** | NM_003073 |
| **PALB2** | NM_024675 | **RAF1** | NM_002880 | **SMO** | NM_005631 |
| **PBRM1** | NM_018313 | **RB1** | NM_000321 | **SRC** | NM_005417 |
| **PDGFA** | NM_033023 | **RET** | NM_020975 | **STK11** | NM_000455 |
| **PDGFB** | NM_002608 | **ROR1** | NM_005012 | **TEK** | NM_000459 |
| **PDGFRA** | NM_006206 | **ROR2** | NM_004560 | **TIE1** | NM_005424 |
| **PDGFRB** | NM_002609 | **ROS1** | NM_002944 | **TP53** | NM_000546 |
| **PIK3CA** | NM_006218 | **RPTOR** | NM_020761 | **TSC1** | NM_001162427 |
| **PIK3R1** | NM_181523 | **RYK** | NM_002958 | **TSC2** | NM_000548 |
| **PMS2** | NM_000535 | **SDHAF2** | NM_017841 | **TYRO3** | NM_006293 |
| **POLD1** | NM_001308632 | **SDHB** | NM_003000 | **VHL** | NM_000551 |

1. Tredan, O., et al., *Molecular screening program to select molecular-based recommended therapies for metastatic cancer patients: analysis from the ProfiLER trial.* Ann Oncol, 2019. **30**(5): p. 757-765.
